# Characteristics, risk factors and outcomes of community-acquired acute kidney injury in the elderly: a prospective tertiary hospital study, Egypt

**DOI:** 10.4314/ahs.v22i2.40

**Published:** 2022-06

**Authors:** Rasha Samir Shemiesa, Mostafa Abdelsalama, Sherouk Salah Elnagara, Ashraf Hussein Mohameda, Nagy Sayed-Ahmeda, Mona Tawfika

**Affiliations:** Mansoura Nephrology and Dialysis unit, Mansoura University, Faculty of Medicine, El Gomhurria st, Mansoura, Egypt, Zip code: 35516

**Keywords:** Chronic Kidney Disease, Community Acquired Acute Kidney Injury, Dehydration, Elderly, Hypertension

## Abstract

**Background:**

Acute Kidney Injury (AKI) is a public health problem. Elderly present a greater predisposition to the development of AKI, either due to kidney senility, or due to high prevalence of comorbidities and polypharmacy. Considering the scarcity of studies on AKI in the elderly particularly in developing countries, this study emphasizes on the pattern and outcome of AKI in the Egyptian elderly.

**Objective:**

To analyze the demographics, risk factors and outcomes of Acute Kidney Injury (AKI) in the Egyptian elderly.

**Methods:**

A total of 199 patients were included over one year and were divided into two groups; group I (79 elderly patients) and group II (120 non-elderly patients). The two groups were compared regarding demographics, risk factors and major outcomes including patient and renal survival.

**Results:**

Elderly patients showed a higher prevalence of Diabetes Mellitus and chronic kidney disease (p=0.004 and 0.005 respectively). Pre-renal causes of AKI principally dehydration represented the major risk factor (p=0.003). Sepsis and hypertension predicted mortality in the elderly (p=0.001 and 0.035 respectively).

**Conclusion:**

In our locality; the elderly is highly vulnerable to AKI. Pre-renal causes principally dehydration represent the main triggers of AKI. Sepsis and hypertension contribute to mortality in this population. Preventive strategies are crucial not only in the hospital but also at home.

## Introduction

Acute kidney injury (AKI) is a public health problem, characterized by a sudden renal dysfunction with consequent increase of nitrogenous products and acid-base disturbances. The overall mortality of AKI is still high in spite of all the therapeutic developments that have taken place in the past few decades^1^,^2^. AKI is frequently encountered in the elderly^3^. The kidneys, as well as the rest of the body, get old. This comprises glomerulosclerosis, interstitial fibrosis, tubular atrophy as well as vascular degenerative changes with consequent senile nephrosclerosis^4^–^6^. Furthermore, numerous conditions besides ageing predispose a patient to AKI, including high prevalence of polypharmacy and comorbidities, for instance, diabetes mellitus, chronic kidney disease, hypertension, and cardiovascular disease. Earlier epidemiologic studies revealed that AKI is increasingly prevalent among elderly people and that there exists an age-dependent association between ageing and AKI^7^. This may be also due to advances in the health area which has led to an increase in life expectancy, hence an increase in the deand for health services^6^. AKI can be classified based on the underlying pathophysiology into prerenal, intrinsic renal injury, and postrenal obstruction, among which, prerenal causes are emphasized. This comprises both reduction in the absolute volume (volume depletion) and the relative volume (cirrhosis and heart failure)^8^. The most prevalent type of AKI is conflicting especially between community and hospital acquired cases. Some authors described prerenal AKI as the most prevalent form^2^, while others stated that intrinsic renal acute tubular necrosis (ATN) was responsible for >50% of AKI cases in hospitalized patients and >76% of AKI cases in intensive care unit (ICU) patients^4^,^9^. Long-term recovery is also less likely, and it is believed that AKI in elderly more often results in chronic kidney disease (CKD)^10^. Management of AKI in elderly generally follows the same principles as for the general population. However, the decision to initiate dialysis in the very elderly with multiple comorbidities may be difficult. Understanding of the greater vulnerability to AKI in the elderly is essential to develop new therapies aiming at mitigating the risk of AKI. Considering the scarcity of studies on AKI in this population, this study emphasizes on the pattern and outcome of AKI in elderly.

## Materials & Methods

### Study design, inclusion, and exclusion criteria

This is a prospective observational study that was conducted at Mansoura nephrology and dialysis unit (MNDU), Mansoura University, over one year. During the study period, we included all patients presented to our unit at the time of admission with impaired kidney function (community-acquired Acute Kidney Injury). They were assigned as having AKI according to the urine output and creatinine criteria of KDIGO (2012); namely, an increase in SCr ≥0.3 mg/Dl (≥26.5 µmol/L) within 48 h; or an increase in SCr to ≥1.5 times baseline, which is known or presumedto have occurred within the prior 7 days or a urine volume of <0.5 Ml/kg/h for 6 h^11^. Patients less than or equal to eighteen years old or those with hospital-acquired AKI were excluded from the study. Participants included in the study comprised of patients with:

1. High serum creatinine at the time of admission with normal basal serum creatinine
2. High serum creatinine at the time of hospitalization followed by a return to baseline values or improved by 50%.
3. Elevated serum creatinine at the time of admission with the absence of history suggestive of CKD, and with normal sonographic kidney examination.
4. Sudden upsurge of serum creatinine by 50% or more in CKD patients (stages 1–4 CKD).

Baseline serum creatinine, if available, was defined as the lowest serum creatinine value that is closest to time of hospital admission and within one year.

## Methodology

A thorough history taking was performed (including a history of diabetes mellitus (DM), hypertension, CKD, chronic liver disease (CLD), cardiovascular disease (CVD), urinary calcular disease, malignancy, autoimmune disorders, drug intake, preceding infection, bleeding, fluid loss, diminished fluid intake or urine output, ...etc.). Physical examination (especially signs of uremia, dehydration, shock, and sepsis) and laboratory assessment (including urine analysis, Complete blood count (CBC), liver function tests, serum creatinine, Arterial blood gases (ABG), and serum calcium) were also done.

The renal sonographic examination was undertaken to evaluate the presence of CKD. Abnormalities in kidney size, echogenicity or other associated problems like urinary tract obstruction were noted. Additionally, added workup was done in selected cases when needed (e.g., immunological tests and renal biopsy) according to the vision of treating physician, and management protocols. Patients were divided according to their age into two groups (elderly and non- elderly), and the age cutoff in the elderly group was considered 65 years or more^12^. The cause of AKI was further classified into pre-renal, intrinsic renal and post-renal based on the physical, laboratory and radiological assessments.

During hospital admission, both groups were regularly assessed by a specialized nephrologist for evaluation of renal function, renal recovery, and the possible need for renal replacement therapy (RRT) initiation. Hemodialysis was initiated according to the local working policy. Hemodialysis was prescribed for patients who suffered from life-threatening uremic complications (like electrolyte or acid-base disorders not responding to medical treatment, severe volume overload, uremic pericarditis, uremic encephalopathy, ...etc), or for those with a serum creatinine of more than 8 mg/dl with the absence of renal recovery. The study included two outcomes: primary outcome, which was all-cause hospital mortality and secondary outcome, which was renal recovery at the time of discharge and for three months. Patients were assigned according to renal recovery outcome^13^ into:

1. Completely recovered: who had serum creatinine at discharge returned to within baseline.
2. Partially recovered: who failed to achieve complete recovery but not requiring renal replacement therapy.
3. Unrecovered: who had no improvement in serum creatinine or remained on dialysis at the time of discharge and after 3 months.

### Patients' consent and ethical consideration

All patients were informed about the study protocol, and methodology and informed consent was obtained prior to enrollment. The studied population was previously investigated^2^. The study was reviewed and approved by the local ethical committee of institutional board review, Approval number: MS/15.06.47.

### Statistical analysis

Data were collected, revised, verified then edited on a personal computer. The statistical analysis was performed using SPSS (Statistical Package of Social Sciences) version 21 for Windows (SPSS, Inc., Chicago, IL, USA).

The normality of data was first tested with Shapiro-wilk test or Kolmogorov Smirnov test when appropriate. Qualitative data were described using number and percent (n, %) and were compared using Fisher exact test or Chi-square test when appropriate. Continuous variables were presented as mean ± standard deviation (SD) for parametric data, and as Median (min-max) for non-parametric data. Unpaired data were compared using Student t-test (for parametric variables) and Mann-Whitney test (for non-parametric variables) with the least significance difference (LSD) between groups at p <0.05. Multivariate logistic regression analysis was used to identify predictors of study outcomes with calculation of odds ratio (OR; and 95% confidence interval, CI). The selected covariates for multivariate regression were the significant factors associated with the outcome that were explored by bivariate analysis.

## Results

During the one-year study period, we evaluated 199 patients presented with community-acquired AKI. All patients were initially assessed, regularly followed-up, and received their medical service until death or for three months in survivors, except for nine patients who lost follow-up for different reasons. Table 1 shows a comparison of demographic, clinical, and laboratory data among both groups.

**Table 1:** Comparison of demographic data, clinical comorbidities, and laboratory findings between elderly and non-elderly groups (n=199)

Variable	Elderly (n=79)	Non-Elderly (n= 120)	P
**Age (year)**	72±6.1	46.1±13.33	<0.0001
**Gender distribution:** **Male n (%)** **Female n (%)**	38(48.1%) 41(51.9%)	58(48.3%) 62(51.7%)	1.000
**DM n (%)**	38 (48.1%)	33(27.5%)	0.004
**HTN n (%)**	37(46.8%)	35(35%)	0.105
**CKD n (%)**	17(21.5%)	9(7.5%)	0.005
**CLD n (%)**	11(13.9%)	13(10.8%)	0.514
**CHF n (%)**	3(3.8%)	3(2.5)	0.683
**Basal serum creatinine (mmol/L)**	0.2 (0.07–0.37)	0.14 (0.07–1.06)	0.122
**Serum creatinine at** **admission (mmol/L)**	0.32(0.08–1.06)	0.3 (0.07–1.59)	0.928
**WBCs (ẋ10^3^/µL)**	9.3(2.7–38000)	9(1.7–31)	0.115
**Hb (gm%)**	9.2±2.4	9.34±2.3	0.945
**Platelets (ẋ10^3^/µl)**	207(8–592)	193(3–658)	0.407
**Blood pH**	7.29±0.1	7.29±0.1	0.620
**HCO_3_ (mmol/L)**	15.1±4.5	15.6±4.2	0.267
**Serum potassium (mmol/L)**	4.8±1.3	4.6±1.2	0.358
**Serum albumin (g/L)**	30.7±7.3	29.4±7	0.167
**Serum bilirubin (umol/L)**	13.68(11.97–256.5)	13.68(11.97–88.92)	0.824
**ALT (IU/ml)**	23(12–499)	22(12–255)	0.324
**AST (IU/ml)**	26(18–632)	25(15–265)	0.199
**Serum calcium (mmol/L)**	2.058±0.25	2.05±0.275	0.855

DM: diabetes mellitus; HTN: hypertension, CKD: chronic kidney disease: CLD: chronic liver disease, CHF: congestive heart failure, WBC: white blood cells, Hb: hemoglobin: PLT: platelets, AST: aspartate aminotransferase, ALT: alanine aminotransferase

**Probability of independent sample t test. Values of its variables are expressed as mean ± SD.**

**Probability of chi-square test. Values of its variables are expressed as n (%).**

**Probability of Mann-Whitney U test. Values of its variables are expressed as median (min-max).**

The elderly group constituted 39.7% of the total sample (n=79/199), with a male to female % ratio of 48.1%:51.9%. Regarding associated co-morbidities, elderly AKI patients showed higher frequencies of diabetes mellitus and CKD (P=0.004, and 0.005), respectively, while hypertension, CLD, and Congestive heart failure frequencies did not statistically differ among both groups. Basal serum creatinine, as well as in-hospital laboratory data, did not much vary among both elderly and non-elderly patients.

Table 2 shows the causes, precipitating factors, and treatment of AKI in the studied sample. Pre-renal causes of AKI were documented more frequently among elderly patients (p=0.019), while intrinsic causes of AKI were more common in non-elderly patients (p=0.027).

**Table 2:** Causes, precipitating factors, and treatment of AKI among both groups (n=199)

Variable	Elderly (n=79)	Non-Elderly (n=120)	P
**Pre-renal AKI** n (%)	62(78.5%)	75(62.5%)	0.019
**Renal AKI** n (%)	12(15.2%)	35(29.2%)	0.027
**Post-renal AKI** n (%)	5(6.3%)	10(8.3%)	0.408
**Dehydration** n (%)	62 (78.5%)	73(60.8%)	0.003
**Infection** n (%)	36(45.6%)	47(39.2%)	0.382
**NSAIDS** n (%)	19(24.1%)	32(26.7%)	0.678
**ACEIs** n (%)	17(21.5%)	15(12.5%)	0.741
**Sepsis** n (%)	12(15.2%)	23(19.2%)	0.115
**Urinary tract obstruction** n (%)	5(6.3%)	7(5.8%)	1.000
**Non-renal Neoplasm** n (%)	4(5.1%)	5(4.2%)	0.743
**Pyelonephritis** n (%)	3(3.8%)	6(5%)	1.000
**Postoperative** n (%)	2(2.5%)	5(4.2%)	0.705
**Chemotherapy associated** n (%)	1(1.3%)	5(4.2%)	0.406
**Multiple myeloma** n (%)	0(0%)	2(1.7%)	0.519
**SLE** n (%)	0(0%)	18(15%)	0.0001
**Contrast nephropathy** n (%)	0(0%)	3(2.5%)	0.278
**Fluid therapy** n (%)	65 (82.3%)	76 (63.3%)	0.004
**Diuretics** n (%)	9 (11.4%)	36 (30%)	0.003
**Hemodialysis treatment** n (%)	23(29.1%)	44(36.7%)	0.287
**Indication of hemodialysis** **Volume overload** n (%) **Electrolyte and metabolic** **disturbances** n (%)	6(7.6%) 18 (22.8%)	10 (8.3%) 37(30.8%)	0.602 0.458
**Hemodialysis dependency at** **discharge** n (%)	11(14.7%)	23(20%)	0.621

NSAIDs: non-steroidal anti-inflammatory drugs, ACEIs: angiotensin converting enzyme inhibitors, SLE: systemic lupus erythematosus.

**Probability of chi-square test. Values of its variables are expressed as n (%)**

Systemic lupus erythematosus (SLE) was diagnosed only in the non-elderly group with a statistically higher significant difference than the elderly group (p=0.0001), on the other hand, dehydration was more frequent in elderly patients (p=0.0003). The frequency of infection, sepsis, drug intake, and urinary obstruction were almost similar among both groups. As regard the treatment options of AKI that were provided to our patients, we noted that the number of patients who needed fluid resuscitation was much higher in the elderly group. At the same time, fluid restriction and diuretic therapy were much more applied in the non-elderly group (p=0.004, and 0.003), respectively. The frequency of hemodialysis treatment was not statistically different among both groups. Seventeen percent of our patients were discharged on dialysis, 5% of them were among the elderly group.

Out of 190 patients who completed the study follow-up, death was reported in 25 patients (13.2%), with no frequency dominance in either group. The frequencies of renal recovery patterns were comparable in both elderly and non-elderly groups, (Table 3).

**Table 3:** Comparison of survival and renal outcome between elderly and non-elderly groups (n= 190/199 patients; 9 patients were lost follow-up)

Variable	Elderly (n=75)	Non-Elderly (n=115)	P
Mortality n (%)	12(16%)	13(11.3%)	0.235
Partial renal recovery n (%)	45(60%)	52(45.2%)	0.054
Complete renal recovery n (%)	6(8%)	11(9.6%)	0.799
Non-recovery n (%)	24(32%)	52(45.2%)	0.072

Probability of chi-square test. Values of its variables are expressed as n (%)

Among elderly group, patients with preexisting CKD, in comparison to those without CKD, showed statistically non-significant differences as regard clinical data, risk factors for AKI, and mortality outcome, (Table 4). However, Elderly patients without CKD showed statistically significant higher ALT level and higher frequencies of infection episodes than patients with CKD (p=0.004, and 0.008) respectively

**Table 4:** Comparison between elderly without CKD and elderly with CKD patients

Variable	Elderly without CKD (n=62)	Elderly with CKD (n=17)	P
**Age (year)**	70(65–86)	70(65–88)	0.618
**Gender distribution** **Male n (%)** **Female n (%)**	31(505) 31(50%)	7(41.2%) 10(58.8%)	0.591
**HTN n (%)**	29(46.8%)	8(47.1%)	0.599
**DM n (%)**	30(48.4%)	8(47.1%)	0.571
**CLD n (%)**	9(14.5%)	2(11.8%)	1.000
**CHF n (%)**	2(3.2%)	1(5.9%)	0.522
**Serum albumin (g/L)**	30.9 ±7.47	29.5±5.9	0.429
**Serum bilirubin (umol/L)**	0.8(0.6–15)	0.8(0.8–4.7)	0.298
**ALT (IU/ml)**	24(12–499)	20(20–57)	0.004
**AST (IU/ml)**	27(18–632)	25(20–78)	0.671
**Serum calcium (mmol/L)**	8.35(6.5–10.4)	8.2(4.5–10/1)	0.863
**Dehydration n (%)**	50(80.6%)	14(82.4%)	1.000
**Infection n (%)**	33(53.2%)	3(17.6%)	0.008
**NSAIDS n (%)**	16(25.8%)	3(17.6%)	0.365
**Sepsis n (%)**	11(17.7%)	1(5.9%)	0.211
**ACEIs n (%)**	12(19.4%)	5(29.4%)	0.279
**Shock n (%)**	4(6.5%)	1(5.9%)	0.708
**Mortality n (%)**	11(19%)	1(5.9%)	0.277

CKD: chronic kidney disease: DM: diabetes mellitus; HTN: hypertension, CLD: chronic liver disease, CHF: congestive heart failure, WBC: white blood cells, Hb: hemoglobin: PLT: platelets, AST: aspartate aminotransferase, ALT: alanine aminotransferase NSAIDs: non-steroidal anti-inflammatory drugs, ACEIs: angiotensin converting enzyme inhibitors.

**Probability of independent sample t test. Values of its variables are expressed as mean ± SD.**

**Probability of chi-square test. Values of its variables are expressed as n (%).**

**Probability of Mann-Whitney U test. Values of its variables are expressed as median (min-max).**

Further analysis was applied for the elderly group according to the occurrence of mortality, and a comparison between dead and alive patients was made. Death was documented in 12/75(16%) of elderly patients. Non-survivors showed a statistically higher frequency of sepsis and higher values of alanine aminotransferase than survivors. At the same time, the remaining clinical and laboratory variables did not statistically differ among both groups, (Table 5). Although hypertension was found in 9/12 (76%) of non-survivors versus 26 (41%) of survivors, the probability of the applied analysis did not exceed the level of statistical significance (p=0.055), which may suggest a clinical rather than a statistical relevance. Multivariate logistic regression analysis showed that presence of hypertension and sepsis in elderly group were the most significant predictors of mortality, however this analysis could not explore any of renal recovery predictors among such group of patients, (Table 6a & 6b) respectively.

**Table 5:** Comparison between non-survivors and survivor groups of elderly patients, n=75

Variable	Non-survivors (n=12)	Survivors (n=63)	P
**Age (year)**	71.6±6.4	71.9±6	0.980
**Gender distribution** **Male n (%)** **Female n (%)**	5 (41.67%) 7 (58.33%)	29 (46%) 34 (54%)	0.430
**HTN n (%)**	9 (76%)	26 (41%)	0.055
**DM n (%)**	5 (41.67%)	32 (50.8%)	0.750
**CLD n (%)**	3 (25%)	7 (11.1%)	0.200
**CKD n (%)**	1 (8.3%)	16 (25.4%)	0.280
**CHF n (%)**	1 (8.3%)	2 (3.2%)	0.400
**Serum albumin (g/L)**	32±10	31±9	0.896
**Serum bilirubin (umol/L)**	13.68 (13.68–256.5)	13.68(13.68–88.92)	0.870
**ALT (IU/ml)**	32 (21–107)	23(12–499)	0.043
**AST (IU/ml)**	34 (20–152)	26(18–632)	0.229
**Serum calcium (mmol/L)**	2.06±0.2	2.05±0.275	0.896
**Dehydration n (%)**	9 (75%)	51 (81%)	0.697
**Infection n (%)**	8 (66.7%)	25 (39.7%)	0.115
**NSAIDS n (%)**	2 (16.7%)	17 (27%)	0.719
**Sepsis n (%)**	6 (50%)	5 (8%)	0.001
**ACEIs n (%)**	0	17 (27%)	0.057
**Non-renal Neoplasm n (%)**	0	3 (4.8%)	1.000
**Postoperative n (%)**	0	2 (3.2%)	1.000

DM: diabetes mellitus; HTN: hypertension, CKD: chronic kidney disease: CLD: chronic liver disease, CHF: congestive heart failure, WBC: white blood cells, Hb: hemoglobin: PLT: platelets, AST: aspartate aminotransferase, ALT: alanine aminotransferase NSAIDs: non-steroidal anti-inflammatory drugs, ACEIs: angiotensin converting enzyme inhibitors.

**Probability of independent sample t test. Values of its variables are expressed as mean ± SD.**

**Probability of chi-square test. Values of its variables are expressed as n (%).**

**Probability of Mann-Whitney U test. Values of its variables are expressed as median (min-max).**

**Table 6.a:** Logistic regression of mortality predictors among elderly group

predictor	B	S.E.	Wald	df	Sig.	Expected (B)	95% C.I.	
							Lower	Upper
Age	.000	.061	.000	1	.995	1.000	.888	1.126
Hypertension	1.888	.894	4.456	1	.035	6.605	1.145	38.121
Sepsis	2.854	.896	10.142	1	.001	17.355	2.997	100.504
Constant	3.448-	4.425	.607	1	.436	.032		

**Table 6.b:** Logistic regression analysis of renal recovery predictors among elderly group

Predictor	B	S.E.	Wald	df	Sig.	Expected (B)	95% C.I.	
							Lower	Upper
Number of dialysis sessions during hospital admission	1.180-	.674	3.063	1	.080	.307	.082	1.152
Creatinine at dialysis initiation	.046-	.168	.076	1	.782	.955	.687	1.326
Fluid restriction	.173-	1.945	.008	1	.929	.841	.019	38.037
Blood platelet	.011	.006	3.310	1	.069	1.011	.999	1.024
Constant	1.518	2.196	.478	1	.489	4.563		

## Discussion

The elderly is greatly predisposed to AKI, either due to frailty, kidney senility, or because of the high prevalence of comorbidities^6^. The incidence of community-acquired AKI and its impact on patient's short and long-term outcomes are largely unknown^14^. There has been a substantial increase in the rates of AKI in the elderly over the past decade^15^. In this study, we evaluated 199 patients presenting with community-acquired AKI. Among those, elderly patients constituted 39.7% of the total sample with a male to female ratio of 48.1%: 51.9%. Community-acquired-AKI (CA-AKI) was defined as AKI developing outside the hospital due, in most cases, to pre-renal causes^16^. Elderly AKI patients in the present study showed higher frequencies of diabetes mellitus and CKD. In general, about 20%–30% of the elderly has DM, therefor, are at risk of developing diabetic nephropathy 17 and/or AKI especially dialysis requiring AKI^18^. CKD may increase the vulnerability of the elderly to AKI because of decreased renal functional reserve^5^. In a parallel study from Tunisia, diabetes and hypertension were the most frequently reported comorbidities seen in about half of the studied patients^19^. In the present study, Pre-renal causes of AKI were documented more frequently among elderly patients, while intrinsic causes of AKI were more common in non-elderly patients, particularly patients with lupus nephritis. Dehydration appears to be the greatest risk factor for AKI in elderly patients included in the present study. Earlier studies showed a greater predisposition of the elderly to AKI triggered by drugs, contrast agents, community acquired infections for instance; community acquired pneumonia when compared to non-elderly patients^20^–^22^. In a Spanish cohort, the incidence of AKI in patients older than 70 years was 3.5-times higher than that in younger patients^23^. In a subsequent study, patients aged >80 years had a 5-times higher risk of AKI than the general population^24^. Elderly patients (≥ 65 years) had 10 times higher incidence rate of AKI in comparison to patients < 65 years in another cohort^25^. In Africa, as there are no consistent statistics about AKI, the Global Snapshot Study adopted by the International Society of Nephrology has determined infections and dehydration as leading causes of AKI^26^, both of which can be more pronounced in the elderly. In an Ivory Coast study, the etiologies of AKI were principally infections and tumors while the main causes of death were principally sepsis and cardiovascular disease^1^. Infections and hypertension were the principal causes for hospital acquired AKI in another African cohort^27^. Elderly patients with underlying chronic kidney disease (CKD) or heart failure are at increased risk for developing AKI when using non-steroidal-anti-inflammatory drugs (NSAIDs), diuretics, and renin-angiotensin-aldosterone-system (RAAS) inhibitors during periods of fluid loss^28^,^22^. Dialysis requiring AKI represents another major health burden. There is a reported annual increase in the incidence of dialysis-requiring AKI of about 10 % through the past decade^29^. It has been thought that patients with CA-AKI can withstand more severe AKI compared to patients with hospital acquired-AKI^30^. In the current study, 29% of elderly AKI patients required dialysis during their hospital admission, of those 14.7% became dialysis dependent. It has been previously reported that 18.9% of elderly patients with AKI progress to dialysis treatment^31^. In the study conducted by Anadkat and Thanki, 50% of the elderly patients required dialysis which was significantly less than did the younger population (91%)^22^. Renal recovery is unlikely in this population, the risk of non-recovery of kidney function is significantly greater in patients older than 65 years^32^. The risk of end stage kidney disease increases significantly among the eldery following AKI episodes^33^. Earlier reports revealed that as about 28% of elderly patients developed CKD following an episode of AKI^4^. The percentage of elderly patients who did not recover their renal function is 32% in the present study with no statistical difference between them and the younger patients. While the reported short-term mortality rates were variable among studies, there exists an agreement on the higher mortality of elderly patients with AKI compared to other age groups. Mortality rates vary depending on the cause of AKI in the elderly, being approximately 35% in patients with pre-renal AKI, 64% (oliguric 76%, non-oliguric 50%) in patients with intrinsic renal AKI, and 40% in patients with post-renal AKI^34^. In-hospital mortality was evaluated in a large cohort of elderly Italian subjects diagnosed with AKI and was found to affect more than a quarter of the investigated population^35^.

The present study showed in-hospital mortality of 16% of the elderly group, with sepsis appears to be the main predictor of death among non-survivors. Elderly people are more susceptible to sepsis. Previous reports showed that the increase in sepsis mortality rates was the greatest among the very elderly^36^,^37^. Sepsis related AKI was associated with higher risk of in-hospital mortality and a longer hospital stay compared to AKI due to any other causes^38^. The degree of renal recovery following an episode of AKI may substantially affect the long-term survival of patients, particularly in patients with sepsis related AKI^39^. In sepsis, numerous factors may contribute to persistent tissue hypoxia, inflammation, and fibrosis leading to delayed recovery and progression to CKD^40^. Hypertension is shown as a significant predictor of mortality in the present study. Numerous recent studies indicate that AKI also significantly increases the risk of hypertension, cardiovascular disease, and mortality in those patients who survive AKI^41^,^42^. Although previous studies highlighted age, comorbidity including CKD and higher severity of AKI as the main risk factors for non-recovery of the kidney function^43^, the present study could not explore any of renal recovery predictors.

## Conclusion

Finally, we can conclude that in our locality, community-acquired AKI (secondary mainly to dehydration) is a major health problem among elderly patients with a high impact on morbidity and mortality. Sepsis and hypertension contribute to mortality in this population. Preventive strategies are clearly important and should focus on combinations of precipitating factors not only in the hospital but also at home. The scarcity of data and lack of consensus in the epidemiology of AKI in the elderly warrants further studies assessing the precipitating factors and preventive strategies.

We admit two important limits of our study; first, a larger sample size may be more informative. Second, drug induced AKI is not entirely evaluated in this study since a uniform standard for grading the effect of nephrotoxic drugs was not available.

## Declarations

On behalf of all authors, the corresponding author states that neither the manuscript nor any significant part of it is under consideration for publication elsewhere.

## Abbreviations

CBC: Complete Blood Count
ABG: Arterial Blood Gases
CLD: Chronic Liver Disease
CVD: Cardiovascular Disease
CA-AKI: Community Acquired AKI
